# Supplementary figures and images for: Structural Neural Connectivity Analysis in Zebrafish With Restricted Anterograde Transneuronal Viral Labeling and Quantitative Brain Mapping
Source: Front Neural Circuits. 2020 Jan 23;13:85. doi: 10.3389/fncir.2019.00085 (PMC6989443; doi:10.3389/fncir.2019.00085)

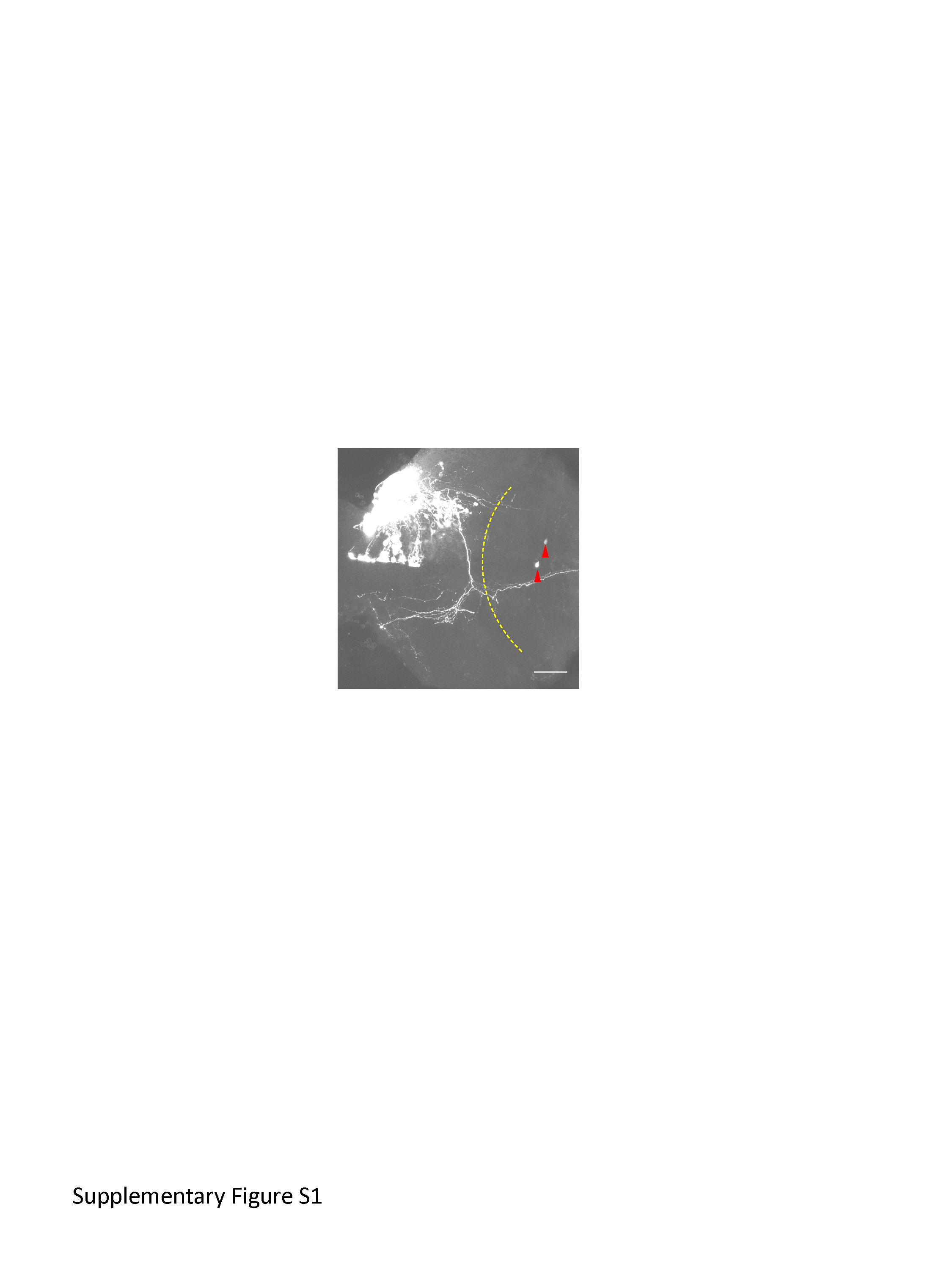

Supplement: Supplementary Figure S1 — Examples of third-order neuron labeling. Red arrowheads indicate third-order neurons labeled in the hindbrain by TRAS. Yellow dash line marks the border of mesencephalon and cerebellum. Scale bar: 50 μm. [file Image_1.TIF]

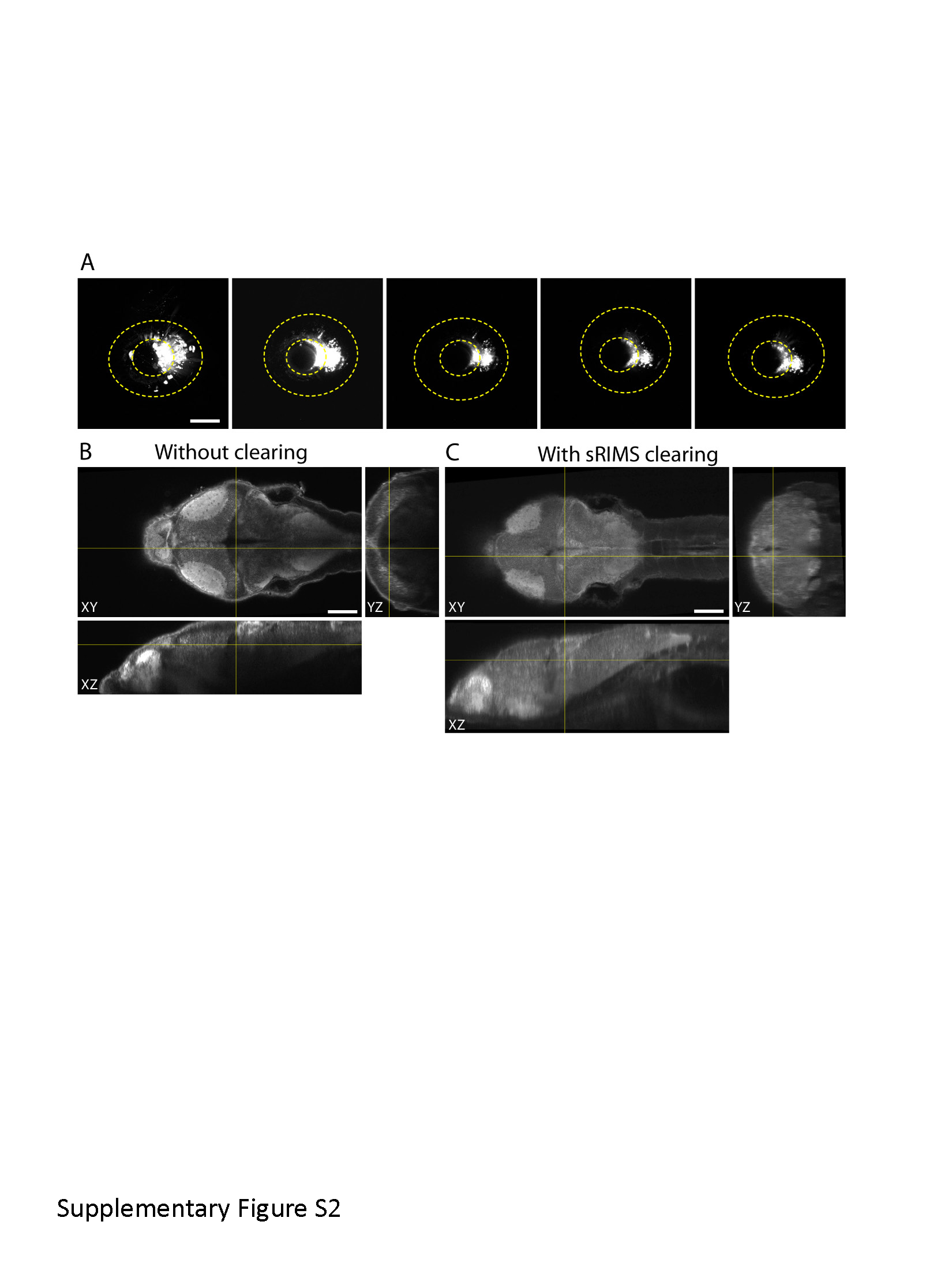

Supplement: Supplementary Figure S2 — Retina injection and tissue clearing. (A) Examples of RFP expression in the retina of unpigmented (PTU-treated) animals 3 days after virus injection into the temporal retina. Images are lateral views, with rostral side to the left and dorsal side to the top. Yellow dashed lines outline the eye (outer oval) and lens (inner oval). (B,C) Tissue clearing with sRIMS solution. Whole-mount ERK1/2 immunolabeling without (B) or with sRIMS clearing (C). Orthogonal views (XY, XZ, and YZ) of confocal image stacks are shown, centered just bellowed the cerebellum (intersect of yellow lines). Ventral structures are not visible without sRIMS clearing. Scale bars are 100 μm. [file Image_2.TIF]

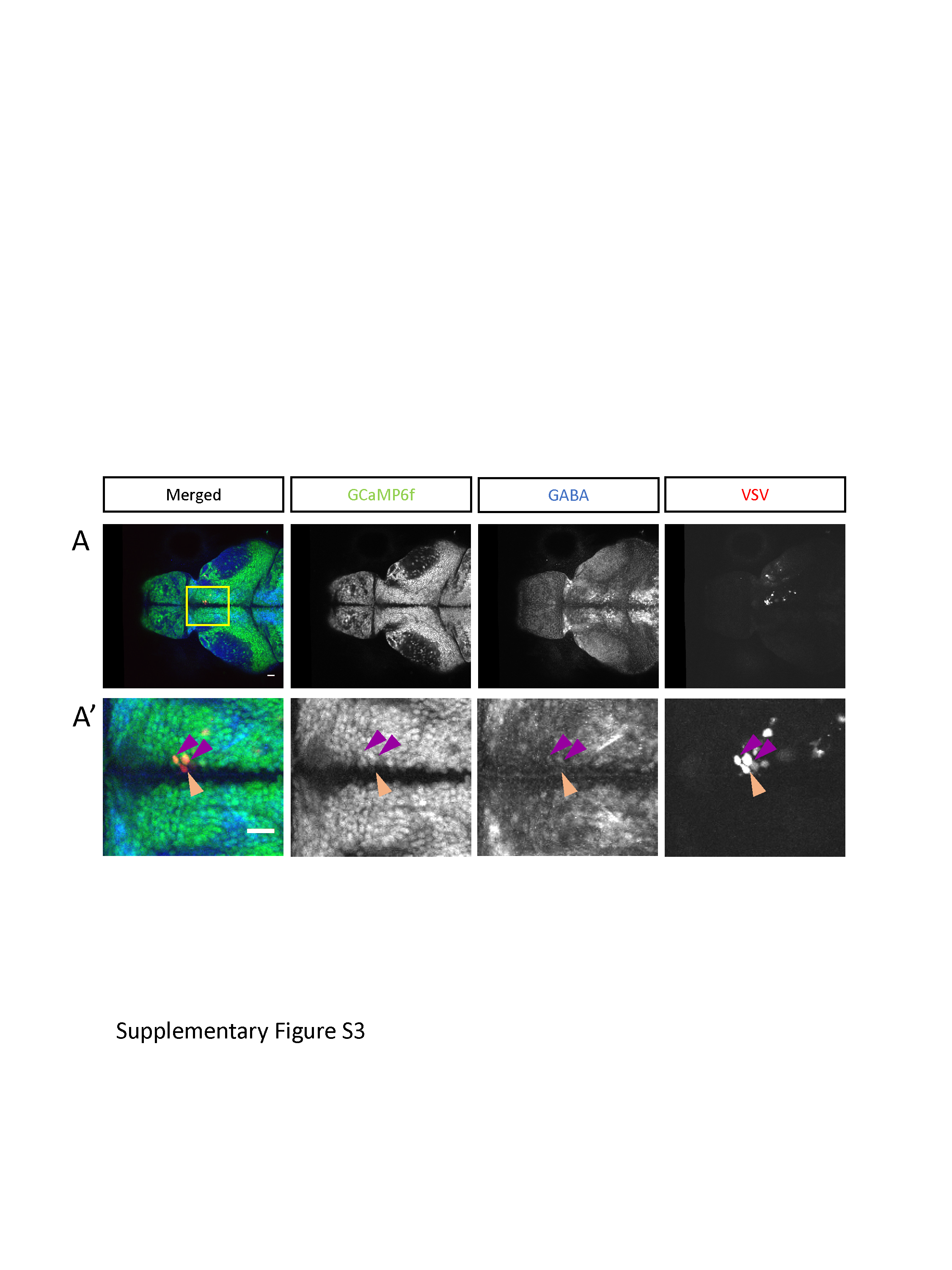

Supplement: Supplementary Figure S3 — TRAS labeling of GCaMP6f positive and negative cells. A single confocal imaging plane is shown, with merged, GCaMP6f, GABA, and VSVΔG channels as indicated. Boxed area in (A) is shown in higher magnification in (A'). The purple arrowheads mark two GCaMP6f+/GABA+/VSVΔG+ inhibitory neurons. The orange arrowhead marks a GCaMP6f–/GABA–/VSVΔG+ cell. Scale bars are 100 μm. [file Image_3.TIF]

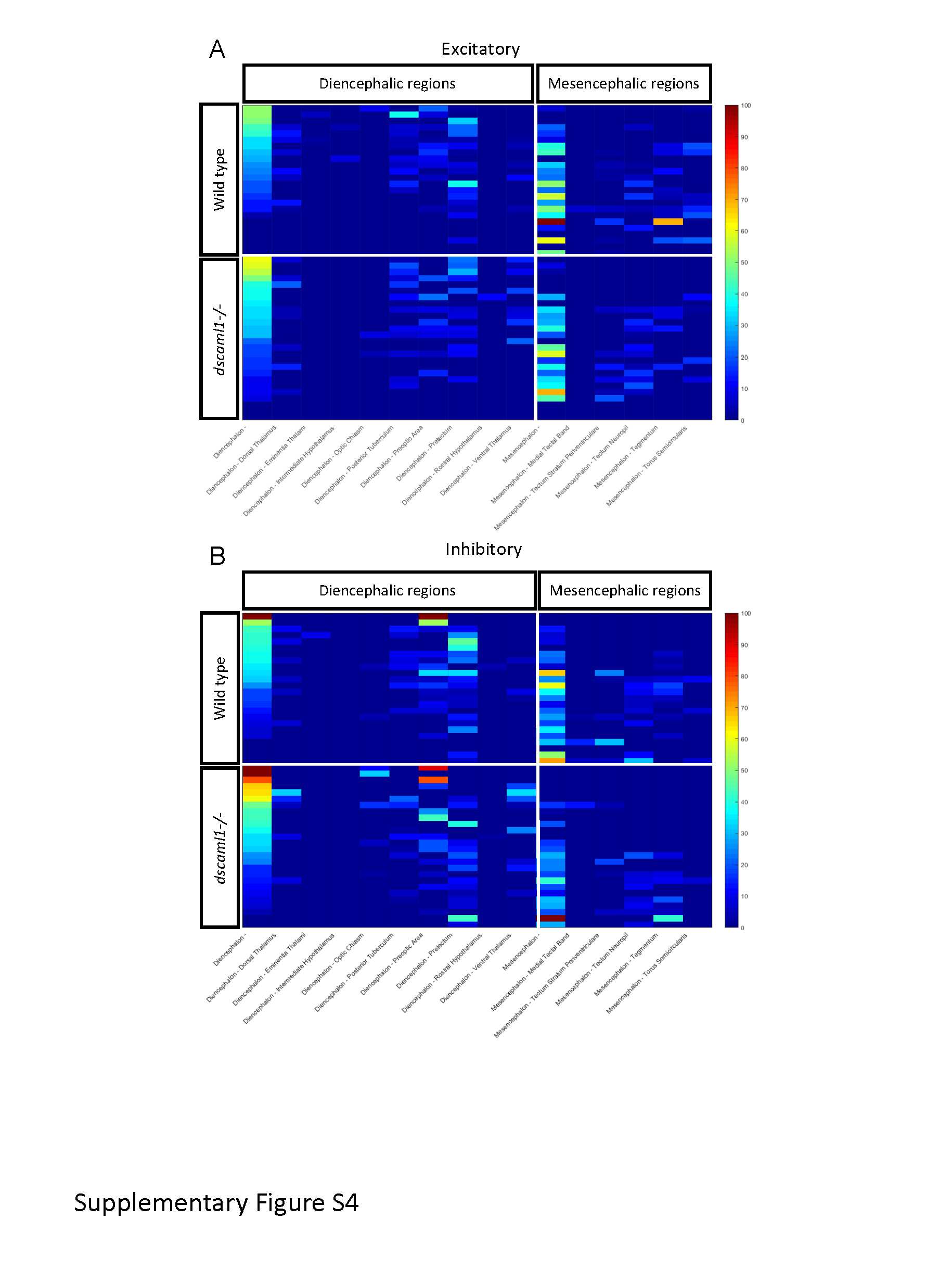

Supplement: Supplementary Figure S4 — Signals detected via Z-brain in wild-type and dscaml1-/- fish. (A,B) Heat map of normalized signals in major anatomical regions from Z-brain showing normalized signals detected within each sample used for analysis, wild-type (n = 24) and dscaml1-/- (n = 26), and their corresponding neuron types (A) is excitatory while (B) is inhibitory. All signals were descended aligning to Diencephalon. [file Image_4.TIF]
